# Brain organoid as a model to study the role of mitochondria in neurodevelopmental disorders: achievements and weaknesses

**DOI:** 10.3389/fncel.2024.1403734

**Published:** 2024-06-24

**Authors:** Raquel Coronel, Enrique García-Moreno, Emilio Siendones, Maria J. Barrero, Beatriz Martínez-Delgado, Carlos Santos-Ocaña, Isabel Liste, M. V. Cascajo-Almenara

**Affiliations:** ^1^Neural Regeneration Unit, Functional Unit for Research on Chronic Diseases (UFIEC), National Institute of Health Carlos III (ISCIII), Madrid, Spain; ^2^Department of Systems Biology, Faculty of Medicine and Health Sciences, University of Alcalá (UAH), Alcalá de Henares, Spain; ^3^Andalusian Centre for Developmental Biology, CIBERER, National Institute of Health Carlos III (ISCIII), Pablo de Olavide University-CSIC-JA, Seville, Spain; ^4^Models and Mechanisms Unit, Institute of Rare Diseases Research (IIER), Spanish National Institute of Health Carlos III (ISCIII), Madrid, Spain; ^5^Molecular Genetics Unit, Institute of Rare Diseases Research (IIER), CIBER of Rare Diseases (CIBERER), Institute of Health Carlos III (ISCIII), Madrid, Spain

**Keywords:** organoid, 3D cultures, spheroids, mitochondrial diseases, neurodevelopmental disorders, high-throughput screening, drug repurposing

## Abstract

Mitochondrial diseases are a group of severe pathologies that cause complex neurodegenerative disorders for which, in most cases, no therapy or treatment is available. These organelles are critical regulators of both neurogenesis and homeostasis of the neurological system. Consequently, mitochondrial damage or dysfunction can occur as a cause or consequence of neurodevelopmental or neurodegenerative diseases. As genetic knowledge of neurodevelopmental disorders advances, associations have been identified between genes that encode mitochondrial proteins and neurological symptoms, such as neuropathy, encephalomyopathy, ataxia, seizures, and developmental delays, among others. Understanding how mitochondrial dysfunction can alter these processes is essential in researching rare diseases. Three-dimensional (3D) cell cultures, which self-assemble to form specialized structures composed of different cell types, represent an accessible manner to model organogenesis and neurodevelopmental disorders. In particular, brain organoids are revolutionizing the study of mitochondrial-based neurological diseases since they are organ-specific and model-generated from a patient’s cell, thereby overcoming some of the limitations of traditional animal and cell models. In this review, we have collected which neurological structures and functions recapitulate in the different types of reported brain organoids, focusing on those generated as models of mitochondrial diseases. In addition to advancements in the generation of brain organoids, techniques, and approaches for studying neuronal structures and physiology, drug screening and drug repositioning studies performed in brain organoids with mitochondrial damage and neurodevelopmental disorders have also been reviewed. This scope review will summarize the evidence on limitations in studying the function and dynamics of mitochondria in brain organoids.

## 1 Introduction

Mitochondria are organelles in almost all eukaryotic cells, performing essential functions such as ATP production, calcium signaling, lipid biogenesis, oxidative stress regulation, and apoptosis. Mitochondrial diseases are a complex and heterogeneous group of multisystemic disorders caused by mitochondria or nuclear DNA mutations, which lead to mitochondrial dysfunction or impaired energy production. Tissues with high-energy requirements are affected in mitochondrial pathologies, particularly the nervous system, whose metabolic needs are even higher during embryonic development. Patients develop early-onset neurological manifestations and neurodevelopmental disorders that are currently incurable (Falk, 2010).

The pathophysiological mechanisms underlying most mitochondrial diseases causing neurodevelopmental disorders are poorly understood. The main obstacle for researchers is the availability of suitable neurological models that recapitulate the phenotype of these complex diseases and allow for the successful translation of results to patients.

In this review, we summarize the role of mitochondria in neural function and development and various protocols for brain organoid generation. Finally, we focus on published cerebral organoid models of mitochondrial diseases and neurodevelopmental disorders, exploring their utility for drug screening techniques. We approach this from the perspective of the advantages and disadvantages of these cerebral models for studying this range of diseases.

**FIGURE 1:**
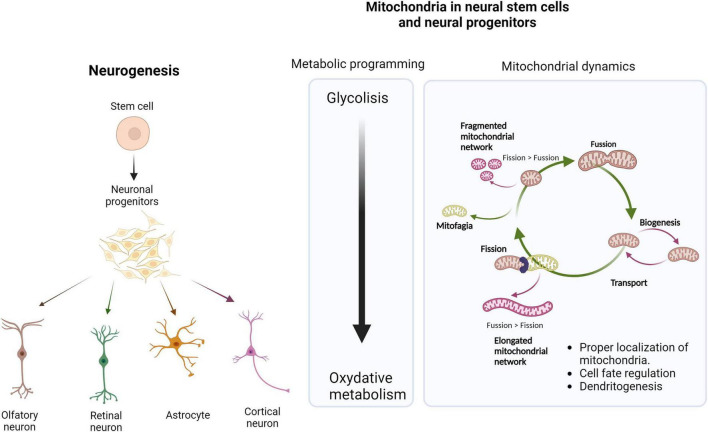
Mitochondria is a central regulator in neurogenesis. Transition from stem cells and neural progenitors to a mature neuronal state is a highly regulated and coordinated process. During neurogenesis, metabolic reprogramming takes place, from NPCs (predominantly glycolytic), to different neuronal lineages dependent on mitochondrial OXPHOS metabolism. Mitochondrial dynamics control the morphology and structure of this organelle, which in addition to enabling metabolic adaptations, are essential for regulating cell fate in neurodevelopment. Created with BioRender.com.

Neurodevelopment refers to the processes involved in the generation and maturation of the nervous system (Gage and Temple, 2013). Neurogenesis or the generation of new neurons from neural stem cells (NSCs) to neural progenitor cells (NPCS), which differentiate into several neural lineages (neurons, astrocytes, and oligodendrocytes), is crucial during the early stages of neurodevelopment, but also in adult tissues (Urbán and Guillemot, 2014; Mira and Morante, 2020; Figure 1). Since the discovery of human embryonic stem cells (hESC), numerous advances have been made, generally focused on cell fate regulation. Several studies have demonstrated a central role of mitochondria in NSCs function and differentiation during embryonic and adult neurogenesis. The role of the mitochondria as energy producers in the form of ATP is one of the factors explaining the association of this organelle with neurodevelopment (Bélanger et al., 2011; Magistretty and Allaman, 2015; Arrázola et al., 2019; Rangaraju et al., 2019). Metabolic programming, defined as significant organ adaptations during the early stages of development, enables the organism to survive nutritional challenges. While embryonic NPCs primarily rely on glycolysis to meet their energy requirements, a metabolic shift toward oxidative phosphorylation (OXPHOS) from stem cells and neural progenitors to mature neurons are critical for proper human neurogenesis and involve specific metabolic regulators, including hexokinase II, lactate dehydrogenase and pyruvate kinase (Wang et al., 2016; Zheng et al., 2016; Figure 1). Evidence shows that mitochondrial function and structure in adult NPCs regulate specific metabolic changes along this neurogenic lineage (Lorenz et al., 2017; Fawal and Davy, 2018; Coelho et al., 2022). In addition, other mitochondrial functions, such as redox signaling, metabolic signaling, and the regulation of gene expression and epigenetics, may impact neurogenesis.

During neurogenesis, mitochondrial dynamics (fission, fusion, and transport) control metabolic adaptations and cell fate (Khacho et al., 2016; Figure 1). The transition to more compromised state cells or NPC is accompanied by mitochondrial fragmentation through NRF2-mediated retrograde signaling. This pathway triggers the suppression of self-renewal and cell differentiation. The transport and proper localization of mitochondria are determining factors in mature neurons. Mitochondria are very dynamic organelles that must travel long distances through the axon, involving microtubules (molecular motors of kinesin and dynein) (Misgeld and Schwarz, 2017; Sheng, 2017; Gumy and Hoogenraad, 2018). The movement of mitochondria, both anterograde and retrograde, is tightly regulated to ensure proper distribution and turnover of these organelles. In mature neurons, mitochondria must be correctly anchored in dendrites and synapses to effectively control the energy supply required for synaptic function and excitability (by local ATP synthesis and Ca buffering) (Rangaraju et al., 2019). In fact, some studies suggest that local Ca^2++^ levels regulate mitochondrial distribution along axons (Greer and Greenberg, 2008; MacAskill et al., 2009; Datta and Jaiswal, 2021). Communication between mitochondria and other organelles, mainly endoplasmic reticulum (ER) and lysosomes, is crucial to regulating various cellular processes through Mitochondria-associated Membranes (MAMs). ER-Mitochondria contact sites play a specific role in mitochondrial fission regulation, calcium metabolism, and lipid transport (Herrera-Cruz and Simmen, 2017; Vallese et al., 2020). Although the importance of mitochondria in the development and function of the nervous system is widely accepted, further studies are required to understand how the mitochondrial networks contribute to dendritogenesis, cell fate, connectivity, and plasticity.

Understanding neurogenesis’s physiopathological mechanisms is essential in neurodevelopmental disorders and mitochondrial dysfunction (Khacho et al., 2019; Oyarzábal et al., 2021). As genetic knowledge of these diseases’ advances, associations between genes encoding mitochondrial proteins and clinical manifestations such as encephalopathy or intellectual delay, among others, are becoming clearer.

Certain mitochondrial disorders differentially affect various brain regions. Like other organs, the nervous system is composed of specific cell types with distinct metabolic needs and characteristics, which can lead to mitochondrial heterogeneity (Rangaraju et al., 2019; Menacho and Prigione, 2020). Evidence suggests that inter- and intra-neuronal mitochondrial diversity may explain the susceptibility of certain neuronal populations to specific mitochondrial disorders. Indeed, fragmented mitochondria have been observed in glycolytic neural stem cells, whereas tubular and interconnected mitochondrial networks are observed in neurons relying on OXPHOS metabolism (O’Brien et al., 2015; Khacho et al., 2016). In comparison to the individual and smaller mitochondria observed in the axons of cortical neurons, elongated and dense mitochondrial networks have been observed in their dendrites (Popov et al., 2005; Chang and Reyolds, 2006; Dickey and Strack, 2011). Various authors have demonstrated differences in the mitochondrial transcriptome and proteome across different populations of neuronal cells (Wynne et al., 2021). For example, there are differences in Mitochondrial Calcium Uniporter (MCU)-mediated calcium buffering capacity between granules and Purkinje cells in the cerebellum, with the former exhibiting greater capacity. This coincided with increased expression of MCU (Fecher et al., 2019). While it is acknowledged that mitochondrial function is associated with the structure and morphology of these organelles, it is important to comprehend how biochemical and structural dysfunctions of mitochondria affect specific cellular populations.

The international neuroscience community has managed, through *in vivo* and *ex vivo* data, to develop an infrastructure for mapping the brain and elucidating the mechanisms involved in neural pathways and their associated disorders. Projects such as Multiomic Human Brain Cell Atlas, Human Brain Project (HBP) (S. LaFee, N. Mlynaryk), EBRAINS Cellular Level Simulation Platform the Blue Brain project (Markram et al., 2015), allow to create computational simulations and *in silico* modeling using AI, neuroinformatics and biocomputing. These computational modeling tools of the brain would allow scientists to validate their results *in silico*.

Traditionally used disease models to study neurodevelopmental pathologies with mitochondrial involvement and their limitations are described below:

• Post-mortem tissues:
  ○ Lack of electrophysiological functions.
  ○ Low frequency of diseases.
• Cell lines and patient-2D cultures: cybrids, primary fibroblast/lymphocytes, neuronal progenitors, or differentiated neurons from patient-iPSC. These cells are divergent from the physiological state of the target tissue/organ, resulting in a lack of cell-cell and cell-matrix interactions and a lack of structural complexity.
• Cancer cell lines mimicking the patient’s mutations for drug screening. Divergent from patient’s physiology and genetic backgrounds.
• Animal models. In many cases, they do not reproduce the neurological phenotype of patients (Huszthy et al., 2012; Inak et al., 2021). The brain of lower mammals lacks many crucial functions that are indicative of higher human cognition.

Mitochondrial functions are tightly controlled and coordinated by nDNA (encodes approximately 1500 mitochondrial proteins) and mtDNA (encodes 13 proteins of the electron transport chain complexes (ETCs), 22 transferents RNA (tRNAs) and 2 ribosomal RNA (rRNAs). The OXPHOS system is composed of five multi-subunit complexes (Complexes I-V; CI-CV), located in the inner mitochondrial membrane. Mitochondria rely on the import of approximately 250 additional nuclear encoded proteins required for OXPHOS function. Each cell contains hundreds to thousands of mtDNA copies, entirely inherited maternally. Unlike the nDNA, the copy number of mtDNA and the proportion of mutated copies (heteroplasmy) varies between cells. Heteroplasmy can be both cell type and mutation specific and the increase in mutated mtDNA copies can lead to diseases. Several factors can alter the degree of heteroplasmy, which can affect embryogenesis and genetic drift across generations. To date, a multitude of genes associated with mitochondrial diseases have been identified in both genomes. The strategies for modeling nDNA or mtDNA mutations are different. Specific and effective methodologies for targeted mutagenesis or mtDNA modification have not yet been established (Hu et al., 2019).

Traditionally, fibroblasts or peripheral blood lymphocytes from patients, and cytoplasmic hybrid (cybrids) cells have been the primary 2D-cell models for studying mitochondrial diseases (Wilkins et al., 2014; Sazonova et al., 2018). Research can be conducted using these primary cells isolated directly from patients and healthy donors or established cell cultures deposited in cell banks. Fibroblasts are the predominant group of cells in the human body, located in almost every tissue and organ. They play a role in numerous functions in connective tissue, being the main source of extracelular matrix (ECM). Fibroblasts are derived from embryonic mesoderm. Primary fibroblasts are easily obtained during muscle or skin biopsy and fibroblasts culture is highly proliferative, providing a renewable source of cells *in vitro* (Hu et al., 2019).

Cytoplasmic hybrid (cybrid) are cell lines developed to eliminate the interference of nuclear background and characterize the physiopathology of mDNA mutations. For that, the nucleus was removed from patient-derived cells such as immortalized lymphocytes or fibroblasts, and cytoplasts were fused with cells lacking mtDNA. Other types of cancer or immortalized cell lines have been used as models of mitochondrial diseases (Wilkins et al., 2014).

However, these cell lines have certain limitations, which are described below:

• Limitations of 2D cultures: (a) genetic and epigenetic abnormalities resulting from long-term *in vitro* culture in such an unphysiological environment; (b) lack of cell-cell and cell-matrix interactions and structural complexity of organ affected by neurological diseases; (c) lack recapitulate mechanisms involved in brain embryonic cells, and (d) lack functional and phenotypic properties of neuron cells and the brain:
  ○ Specific limitations of primary cells lines: (a) short life span; (b) low transfection efficiency (difficulty for gene knockout, knockdown, or editing); (c) in case of patients with mutations in mtDNA, primary cells harboring the same pathogenic variants may exhibit different phenotype resulting from their different nuclear genes; (d) they are not the target cells affected in the patient.
  ○ Specific limitations of cancer or immortalized cell lines: (a) less dependent on oxidative phosphorylation; (b) divergent from patient’s physiology and genetic backgrounds, and (c) disrupt the patient-specific interaction between nDNA and mtDNA.

The specific factors and pathways regulating cellular differentiation, migration, spatial and morphogenic patterns and neurological development, involved in the physiopathology of neurodevelopmental disorders and mitochondriopathies, are not recapitulated in 2D cell cultures. On the other hand, studies and modeling of mitochondrial diseases are complex, mainly due to the complexity of mitochondrial genetics and the interaction between mutations in the mitochondrial genome and nuclear genome and it forces us to explore models that are more physiological and closer to the patient, such as hESC and organoids. The development of 3D cultures and brain organoids derived from patient cells is driving the modeling of such complex and rare diseases, presenting an opportunity to overcome the limitations of 2D cultures and animal models, which are far removed from the patient. However, the coexistence of both 2D and 3D cultures, depending on the research objective, is essential for advancing the study and development of treatments for human diseases. All these limitations and advantages of the 2D models are summarized in Table 1.

**TABLE 1:** Advantages and disadvantages of 2D and 3D-cell cultures as model of neurodevelopmental and neurodegenerative diseases and mitochondrial disorders.

Modeling of neurodevelopmental and neurodegenerative diseases and mitochondrial disorders			
	**2D-cell cultures (primary/immortalized/cancer cells)**		**3D-cell cultures**
**Pros**	Cost effective Easy to manipulate Highly proliferative Well-described and widely used protocols and techniques Reproducible results		Higher resemblance to an embryonic human brain Identical genetic background if generated directly from the patient Less ethical concern than animal/mammalian models Results easier translated to patients
**Cons**	Genetic and epigenetic abnormalities resulting from long-term *in vitro* culture Unphysiological environment No cell-cell and cell-matrix interactions Lack lamination and structural complexity Lack of functional and phenotypic properties of neuron cells and the brain Unable to perform recapitulating mechanisms involved in brain embryonic cells Metabolic discrepancies and mitochondrial dynamics with the target cell/tissue		High-cost Requires precise handling and not easy to culture Sample heterogeneity and lack of reproducibility intra- and inter- laboratory. Low standardization of protocols Differences in the degree of maturity Limited scalability
	**Cancer/immnortalized cells**	**Primary cultures**	
	Less dependent on oxidative phosphorylation Divergent from patient’s physiology and genetic backgrounds Disrupt the patient-specific interaction between nDNA and mtDNA	Short life span Low transfection efficiency Phenotypic differences for the same pathogenic variant in mtDNA in patients with differences in nDNA Not the target cells affected in the patient	

Human pluripotent cells, such as Embryonic Stem Cells (ESCs) and induced Pluripotent Stem Cells (iPSCs), have great potential for human rare disease research. Several authors to differentiate these stem cells have applied previous knowledge from the field of developmental biology (Clevers, 2016). However, thanks to Eiraku and Sasai’s research, in 2012 the first *in vitro* neural organogenesis model systems emerged. These systems were able to replicate not only the cellular differentiation process observed in adherent (monolayer) cultures of stem cells, but also the spatial and morphogenic patterns involved in the three-dimensional (3D) development of certain neural structures (Eiraku and Sasai, 2012). Late, Lancaster and Knoblich (2014) took this approach to a higher level, generating the so-called “cerebral organoids”, which were described as an *in vitro* 3D-culture system capable of generating different brain regions, with a brain-like organization in human development (Lancaster et al., 2013).

Currently, the term “organoid” refers to cellular aggregates derived from primary adult tissues or stem cells capable of self-organizing and forming organotypic structures *in vitro* (Shankaran et al., 2021). Some of the specific brain functions that are critical to be represented in brain organoids to model mitochondrial diseases are mainly neurogenesis, synapsis and function of glial cells (CNS homeostasis maintenance). For instance, it is important that the brain organoids are composed of more than one cell type (1) since some studies affirm that astrocytic mitochondria metabolize long-chain fatty acids more efficiently than neuronal mitochondria (Fecher et al., 2019). This is of interest because fatty acids seem to contribute to the cellular energy metabolism of the brain, although in a lower proportion than glucose (the major oxidative fuel for the brain) (Ebert et al., 2003). On the other hand, it is also essential that brain organoids show specific functions (2), such as synaptic function. For example, in neurons, mitochondria play key roles in energy metabolism and Ca^2+^ regulation, necessary for axonal and dendritic development, axonal regeneration and synaptic function (Rangaraju et al., 2019). Likewise, it is key that brain organoids present a cellular organization similar to that of the brain (3) for proper cellular functionality. For instance, in the brain, it has been described that neurons operate in a strong physical and metabolic association with astrocytes and glial cells, and the dysregulation of this cooperation contributes to the development of brain disease (Bonvento and Bolaños, 2021). In addition, through a mechanism called transmitophagy, the neurons can secrete damaged mitochondria that can be taken up and degraded by adjacent astrocytes (Davis et al., 2014), highlighting the need for adequate cellular and tissue organization in the brain. Since the pathways involved in mitochondrial metabolism, calcium buffering, mitochondrial dynamic (mitochondrial trafficking through the axons, mitophagy, fusion and fission), regulation of apoptosis and oxidative stress are essential to an adequate differentiation, development and maturation of CNS, the recapitulation of all these processes in brain organoids is a major challenge.

**FIGURE 2:**
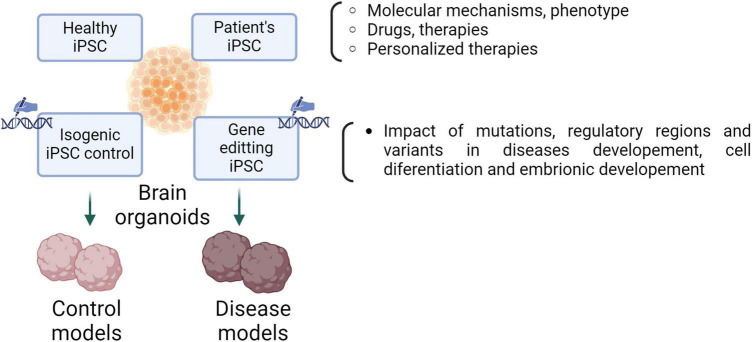
Brain organoids as a model for neurodevelopmental disorders to elucidate the molecular mechanism of the disease, discovery of new drugs and/or therapy. 3D models from patient’s somatic cells and healthy individuals, replicating phenotypic characteristics, allow the study of the molecular mechanisms of pathology, drug screening or personalized therapies. CRISPR techniques applied in iPSCs are used both to induce specific mutations and to correct mutations associated with a specific disease. Thus, brain organoids from isogenic lines enable the investigation of the impact of mutations, regulatory regions and variants on the disease, neural development, and cell differentiation. Created with BioRender.com.

Unlike other types, human brain organoids are usually generated only from human pluripotent stem cells (both hESCs and hiPSCs), mainly due to the low regenerative capacity of the adult human brain (Tang et al., 2022). Pluripotent stem cells can differentiate into more specialized cells derived from the three germ/embryonic layers (endoderm, ectoderm, and mesoderm) (Liu et al., 2020). On the one hand, hESCs are stem cells derived from the human blastocyst’s inner cell mass (ICM). They can be cultured *in vitro* indefinitely and retain the expression of several key epiblast markers, such as the transcription factors Oct4 and Sox2 (Nair and Gongora, 2020; Weatherbee et al., 2021). On the other hand, hiPSCs are stem cells derived from the *in vitro* reprogramming of human fibroblasts. They emerged in 2007 thanks to the research of Yamanaka and Takahashi, who discovered the four transcription factors (Oct4, Sox2, Klf4, and c-Myc, abbreviated as “OSKM”) necessary for human fibroblasts reprogramming (Takahashi et al., 2007). Both hESCs and hiPSCs are two-dimensional (2D) cultures, and they are used in biomedical research as an excellent experimental platform for studying human development, disease modeling, drug screening, and cell therapy (Yang et al., 2021; Figure 2).

Brain organoids recapitulate some features of the fetal human brain, not only at the cellular level but also in structure, tissue organization, and developmental trajectory. Their applications have continued to expand, and protocols for generating human cerebral organoids from ESCs and iPSCs are growing exponentially. Many authors consider human brain organoids to be an excellent model for studying several aspects related to the biology of human brain development (Di Lullo and Kriegstein, 2017; Qian et al., 2019). Furthermore, the possibility of generating human brain organoids from hiPSCs derived from patients (e.g., with a neurodevelopmental disease) allows *in vitro* modeling of several brain disorders (while maintaining the genomic context) (Sidhaye and Knoblich, 2021), which would contribute to the study and understanding of the mechanisms involved in mitochondrial diseases and neurodevelopmental disorders (Soldner et al., 2011).

Studies on how mitochondrial dysfunction affects specific pathways involved in developing and maintaining the nervous system need to be addressed. Neurological 3D models for overcoming the limitations of 2D cultures and animal models are expanding enormously. The ability to replicate, in the laboratory, the early stages of human brain development to understand how mitochondrial dysfunction affects its normal development represents a great hope for researchers and patients of this group of rare diseases. Currently, the challenge is to standardize the protocols for the generation of brain organoids, to automate and scale the processes (from generation to the analysis of large amounts of data), and to efficiently translate results to patients. Cooperation between disciplines, such as neurological development, mitochondrial diseases, and engineering in consortia with private companies, is essential to obtain meaningful and reliable results.

## 2 Brain organoids: methodologies for generation, types and characteristics

### 2.1 Methodologies for generation of brain organoids

The initial work on the differentiation of 2D cultures (Zhang et al., 2001; Chambers et al., 2009a,b; Shi et al., 2012) and the research of Eiraku and Sasai on 3D cultures (Eiraku et al., 2011) have been the basis for the development of protocols for the generation of brain organoids. There are two main protocols or methods for generating brain organoids, resulting in several types of brain organoids. On the one hand, in the unguided protocol, cerebral organoids are generated and differentiated spontaneously from pluripotent stem cells without the need to include extrinsic factors in culture media during their development (Kim et al., 2021). In this case, the resulting organoids contain several brain regions in their structure (Lancaster et al., 2013; Lancaster and Knoblich, 2014; Quadrato et al., 2017). On the other hand, in guided protocols, brain organoids are generated and differentiated from pluripotent stem cells by adding extrinsic and morphogenic factors (called patterning factors) to culture media. Such factors provide the desired regional identity pattern (Kim et al., 2021). In this case, the resulting organoids are specific to a particular brain region (cerebral cortex, hypothalamus, midbrain, etc.) (Paşca et al., 2015; Qian et al., 2016; Lancaster et al., 2017).

Most of these methodologies (guided and unguided) start with the formation of embryoid bodies (EBs) from pluripotent stem cells. These EBs are 3D spherical cellular aggregates that resemble structures from very early events of embryogenesis. Subsequently, EBs are differentiated and maintained in culture until the formation of brain organoids (Benito-Kwiecinski and Lancaster, 2020). The large structural complexity of the brain may lead to a difficult challenge to overcome in unguided protocols, as they are often unable to identically recapitulate all the different brain regions, thus also causing heterogeneity between batches (Kelley and Paşca, 2022). In the case of guided protocols, they usually present greater homogeneity between batches, although the organoids generated only recapitulate a specific brain region, losing the wide structural and regional diversity that characterizes the human brain (Kim et al., 2021). Recently, González-Sastre and Liste have developed and optimized an efficient protocol for the generation of human cerebral organoids that does not involve forced aggregation of EBs but rather directly carries out neural induction of pluripotent stem cells in culture, moving from a 2D to 3D model. They achieve this through the strategic combination of several guided and unguided approaches (previously described in the field), generating cerebral organoids with a correct spatial arrangement and a high homogeneity between batches (González-Sastre et al., 2024). As discussed above, most of the methods described for the generation of brain organoids start with the formation of EBs. However, this process has some limitations, such as marked cellular stress and cell death by apoptosis and anoikis detected during the dissociation process and the forced aggregation of pluripotent stem cells. This fact sometimes causes a high variability between brain organoids generated in different batches and an alteration in their cellular differentiation (Ryu et al., 2024). Therefore, as the authors comment, this new approach of direct transition from 2D to 3D cell culture (achieved only by a strategic combination of patterning factors and avoiding the forced aggregation of pluripotent stem cells into EBs) (González-Sastre et al., 2024) could help to overcome some of the existing limitations in the field of organoids. In addition, the authors state that the protocol developed is capable of generating brain organoids from several human pluripotent stem cell lines, both hESCs and hiPSCs, which show high consistency and reproducibility.

**FIGURE 3:**
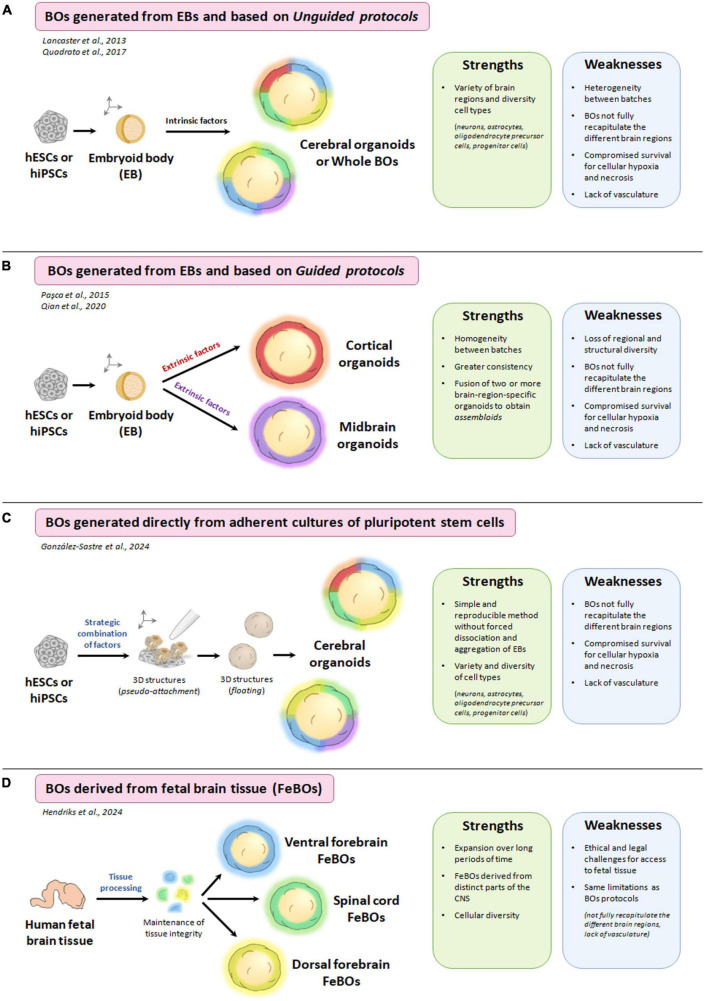
Different methodologies for the generation of brain organoids (BOs) and the strengths and weaknesses of each of them. **(A)** Schematic process for the generation of brain organoids from embryoid bodies (EBs) and based on unguided protocols. **(B)** Schematic process for the generation of brain organoids from EBs and based on guided protocols. **(C)** Schematic process for the generation of brain organoids directly from adherent cultures of pluripotent stem cells. **(D)** Schematic process for the generation of brain organoids derived from fetal brain tissue (FeBOs). Created with BioRender.com.

Another recent and innovative research has been developed by Hendriks et al. (2024), who succeeded in generating brain organoids derived from tissue stem cells instead of pluripotent stem cells (Hendriks et al., 2024). Before the publication of these studies, only the generation of pluripotent stem cells-based brain organoids had been established (due to the low regenerative capacity of adult brain tissue). In this paper, the authors demonstrate the generation of brain organoids derived from human fetal brain tissue (FeBOs) and their maintenance in defined culture conditions over long periods. These FeBOs recapitulate several aspects of cellular heterogeneity and complex organization *in vivo* of the developing human brain (Hendriks et al., 2024), opening an independent avenue to study the human fetal brain in health and disease. A representative scheme where the differences of each methodology for generation of brain organoids are indicated in Figure 3. As described so far, brain organoids (as well as 2D models) are great strategies that provide the opportunity to study neurodevelopment and neurological diseases (including mitochondrial diseases). However, there are still some challenges that limit their usefulness.

Traditional 2D cultures have been the *in vitro* models used for the longest time in biomedical research and have allowed us to advance our biological knowledge. However, conventional 2D cell cultures lack a tissue structure (with a cellular organization and architecture established) and a development trajectory, these being some of the weaknesses compared to more complex models, such as organoids (Qian et al., 2019). In the case of the human brain, which is a very complex organ and practically inaccessible to direct experimentation (particularly during the different stages of neurodevelopment), the generation of several methodologies for obtaining brain organoids has represented a great advance (Kim and Chang, 2023). In addition, as mitochondrial diseases particularly affect tissues and cells that are highly dependent on mitochondrial energy production (such as neuronal, muscle and cardiac cells) (Pfeffer et al., 2013), the use of brain organoids to study the role of mitochondria in neurodevelopmental disorders is a revolutionary strategy. Nevertheless, although brain organoids provide a unique opportunity to model the development and function of the human brain, this novel methodology also has weaknesses.

One limitation is related to the reproducibility of brain organoids generation within batch-to-batch variations (Chiaradia and Lancaster, 2020). This weakness is shared by all the methodologies previously described for the generation of brain organoids, although is most prominent in unguided protocols (with variations in size, morphology, cell composition, cell differentiation and maturation efficiency) (Tanaka et al., 2020). It is important to highlight that the size and morphology of human brain organoids also vary depending on the pluripotent stem cell lines used (Kanton et al., 2019). Therefore, it is essential to have adequate controls for age, genetic background or sex to reveal the pathological phenotype of a specific disease.

On the other hand, some drawbacks that brain organoid protocols collectively share are limited oxygen and nutrient diffusion with consequent necrosis, cellular immaturity, and the absence of certain cell types found in the brain, such as microglia and vascular cells (Chiaradia and Lancaster, 2020). Addressing these weaknesses is essential to improve the reliability and applicability of brain organoid models in mitochondrial and neuroscience research.

Another important limitation of current protocols includes the lack of vasculature in the generated brain organoids that limits the supply of nutrients and oxygen to the organoid core. This causes stress and cell death inside the organoid due to hypoxia and lack of nutrients.

One approach to vascularize the human brain organoids consists of transplanting them into the brains of immunodeficient rodents. This leads to vascularization and better maturation of the transplanted brain organoids (Revah et al., 2022). However, this strategy is difficult to scale up. Another strategy that has been used consists of including cells derived from the vasculature, of mesodermal origin, to the human brain organoids, derived from the ectoderm. It has been seen that brain organoids containing endothelial cells derived from hiPSCs develop structures similar to vessels (Pham et al., 2018). Furthermore, the expression of endothelial transcription factors within human brain organoids promotes neuronal maturation and reduces cell death (Cakir et al., 2019).

Other studies have been based on the co-culture of human umbilical vein endothelial cells (HUVECs) with pluripotent stem cells before neural induction, which generated a vascular system formed by the HUVECs in the brain organoids, called HUVEC-BOs (Shi et al., 2020). These HUVEC-BOs presented a reduction in the expression of hypoxia and cell death markers and were larger than non-vascularized brain organoids. Importantly, HUVEC-BOs recapitulated many aspects of neurogenesis and gliogenesis in human cortical development, had greater functional maturation and synaptic connections, and showed increased expression of P-glycoprotein, an important efflux transporter in brain endothelial cells of the blood-brain barrier (BBB). Furthermore, this vascular system derived from HUVECs managed to integrate with the host vasculature after being transplanted into the mouse cortex (Shi et al., 2020). Another study in 2022 took a similar approach and showed that BBB-like structures were formed in brain organoid-vessel organoid (BO-VO) co-cultures and even showed selective permeability for a BBB-penetrating peptide (Sun et al., 2022). These BO-VO co-cultures also contained cells that resembled microglial cells, the immune cells of the brain.

However, vascularized brain organoids currently show very limited BBB properties, which will likely affect the development of these organoids and restrict their uses in modeling brain disorders. Furthermore, current brain organoids do not have cerebrospinal fluid (CSF), which is secreted by the choroid plexus. The combination of vascularized brain organoids and choroid plexus organoids could be an approach to produce a more complete vasculature in cerebral organoids (Pellegrini et al., 2020). Well vascularized brain organoids will be critical for studying human diseases involving brain endothelial cells or pathogenic processes that occur later in life.

### 2.2 Types of brain organoids and their characteristics

Different types of brain organoids can be obtained depending on the methodological process used during the culture of organoids. Like fetal brain development, during the development of brain organoids, several cell types with a specific regional identity are generated, and this is due to several sequential trajectories of cell differentiation (Kim and Chang, 2023).

Based on previously described, brain organoids obtained from unguided protocols are generally called cerebral organoids or whole brain organoids. Using this approach, pluripotent stem cells have greater freedom of self-organization and differentiate into neuroepithelial structures that exhibit a wide variety of cell lineage identities, from the forebrain, midbrain, and hindbrain to mesoderm (Lancaster et al., 2013; Lancaster and Knoblich, 2014; Quadrato et al., 2017; Uzquiano et al., 2022). Single-cell transcriptomic assays have revealed that these cerebral organoids are characterized by containing neural progenitor cells, inhibitory neurons, excitatory neurons, oligodendrocyte precursor cells, and astrocytes (Quadrato et al., 2017). The primary benefit of cerebral organoids is the ability to model and study interactions between different brain regions and mimic neurodevelopmental diseases due to the presence of multiple cell types in the same environment.

In contrast, brain organoids generated from guided protocols are called brain region-specific organoids. These organoids are created through the appropriate combination of cytokines, small molecules, and/or patterning factors, which direct the differentiation of EBs from pluripotent stem cells toward cells and tissues representative of specific brain regions (Zhang et al., 2001; Qian et al., 2019). Most of the methodologies for generating brain-region-specific organoids began with dual SMAD inhibition, achieved by administering TGF-β and BMP receptor inhibitors (Chambers et al., 2009a,b). This inhibition directs pluripotent stem cells toward a neuroectodermal lineage and enhances the formation of neural epithelium, which contains neural precursor cells (NPCs). Different extrinsic patterning factors can differentiate these NPCs into different cerebral lineages, resulting in the formation of region-specific brain organoids (Susaimanickam et al., 2022). Specifically, several guided differentiation methodologies have been described for obtaining forebrain lineage organoids [as cortical organoids (Paşca et al., 2015; Sloan et al., 2018; Trujillo et al., 2019; Qian et al., 2020; Urresti et al., 2021; Eigenhuis et al., 2023); hippocampal organoids (Sakaguchi et al., 2015; Ciarpella et al., 2023); retinal organoids (Völkner et al., 2016; Fligor et al., 2018; Wahle et al., 2023); thalamic organoids (Xiang et al., 2019; Kiral et al., 2023); hypothalamic organoids (Ozone et al., 2016; Qian et al., 2016; Huang et al., 2021); and choroid plexus organoids (Pellegrini et al., 2020)], midbrain linage organoids (Jo et al., 2016; Smits et al., 2019; Nickels et al., 2020; Smits and Schwamborn, 2020; Sabate-Soler et al., 2022), and hindbrain linage organoids [as cerebellar organoids (Muguruma et al., 2015; Chen et al., 2023; Atamian et al., 2024)]. Guided protocols can also be used to generate two or more brain-region-specific organoids separately and subsequently fuse them, obtaining the “assembloids,” which model the interactions between different brain regions (Jang et al., 2022). For example, assembloids of thalamic and cortical organoids (Xiang et al., 2019), as well as cortico-striatal assembloids (Miura et al., 2020) have been described.

The generation of a variety of brain organoids that mimic subregions of the human brain, including the forebrain, hindbrain, hypothalamus, midbrain, and neocortex, has become a reality, as we described above, overcoming the previous lack of human neurological models (Kofman et al., 2022). It is even possible to combine different organoids from diverse cell populations to develop assembloids and higher-order organ structures (Kanton and Pasça, 2022). However, disease models that recapitulate human mitochondrial dysfunction and neurodevelopmental disorders remain limited.

## 3 Brain organoids as models to study the role of mitochondria in neurodevelopmental diseases

Diseases caused by alterations in mitochondrial DNA (mtDNA) and nuclear DNA (nDNA) have proven challenging to model due to their inherent complexity, particularly the difficulties in manipulating mtDNA (Schon et al., 2020). This hampers the generation of diverse *in vitro* and *in vivo* models. However, recent advancements in iPSCs modeling, particularly with 3D organoids, have provided opportunities to develop innovative models for these diseases. Brain organoids enable the assessment of specific mtDNA and nDNA mutations during neurodevelopment in a human disease context. They can uncover molecular mechanisms involved in pathology and evaluate the efficacy of targeted treatments or gene therapy efficacy *in vitro*. The brain organoids and NPCs generated to date as models of mitochondrial diseases affecting the nervous system are discussed below and summarized in Table 2. Most of these models focus on diseases caused by mutations in mitochondrial DNA.

**TABLE 2:** Cellular models for mitochondrial diseases based on the use of iPSCs.

Syndrome	Genes	Cellular models	Described alterations	Therapies	References
Leigh syndrome (LS)	MT-ATP6 (m.8993T > G) MT-ND3 (m.10191T > C) SURF1 (c.530T > G/c.769G > A)	Cortical brain organoids	NPC dysfunction	Benzafibrate	Galera-Monge et al., 2016; Hattori et al., 2016; Grace et al., 2019; Inak et al., 2021
Mitochondrial Encephalopathy, Lactic Acidosis, and Stroke-like episodes (MELAS)	MT-TL1 (m.3243A > G) MT-TW (m.5541C > T)	Motor neurons NPCs Spinal organoids	Reduced neuronal network activity, motor neurons deficits, defective neuronal differentiation	DAPT	Hämäläinen et al., 2013; Hatakeyama et al., 2015; Klein Gunnewiek et al., 2020; Winanto et al., 2020
Myoclonic Epilepsy and Ragged-Red Fibers (MERRF)	MT-TK (m.8344A > G)	NPCs	Impaired mitochondrial function		Chou et al., 2016
Leber’s Hereditary Optic Neuropathy (LHON)	MT-ND4 (m.11778G > A)	Neurons Retinal ganglion cells	Defective neurite outgrowth, enhanced mitochondrial biogenesis, abnormal mitochondrial transport, disrupted action potential, increased mitophagy and autophagy	Idebenone	Wu et al., 2018; Yang et al., 2019, 2020; Danese et al., 2022
Mitochondrial Neurogastrointestinal Encephalomyopathy (MNGIE)	TYMP	Brain organoids	Unexplored		Pacitti and Bax, 2018
Friedreich’s ataxia (FRDA)	FXN (GAA expansion)	Dorsal root ganglia organoids Neurospheres and neurons	Impaired sensory neuron survival	CRISRP-Cas9 removal of expansion	Hick et al., 2013; Mazzara et al., 2020
Mitochondrial DNA depletion syndrome (MDS)	DGUOK	Liver organoids Hepatocytes	Reduced ATP production, ROS increase	NAC	Guo et al., 2021
Chronic Progressive External Ophthalmoplegia (CPEO)	POLG1	Dopaminergic neuron-containing spheroids	Increased inflammation and glycolysis		Chumarina et al., 2019
Kearns–Sayre Syndrome (KSS)	mtDNA deletions of unknown cause	NPCs	Absence of mtDNA alterations in iPSCs derived from patients’ blood	Cell replacement therapy	Sequiera et al., 2021
Pearson Marrow–Pancreas syndrome (PMPS)	mtDNA deletions of unknown cause	Erythroid cells	Absence of mtDNA alterations in iPSCs derived from patients’ fibroblasts	Cell replacement therapy	Cherry et al., 2013

Disease, affected genes, type of model, patient-specific alterations described in the model and therapies tested or suggested are described. NAC, N-acetylcysteine; NPCs, neural progenitor cells.

### 3.1 Syndromes caused by mtDNA and nDNA point mutations

#### 3.1.1 Leigh syndrome

Leigh syndrome (LS) is a rare inherited neurological disorder that primarily affects infants and young children, caused by mutations in more than 75 genes of both mitochondrial and nuclear DNA (Lee et al., 2020). It is characterized by progressive neurodegeneration, developmental delay, muscle weakness, respiratory problems, optic atrophy, and other symptoms such as seizures and feeding difficulties. These manifestations stem from mitochondrial dysfunction, often caused by mutations in genes related to mitochondrial function, leading to impaired energy production and widespread neurological and metabolic abnormalities. Although LS is characterized by neuronal impairment, the exact mechanisms have been unclear due to a lack of effective model systems. The fact that the syndrome presents a range of severity and variability in its clinical manifestations and a high genetic heterogeneity makes the development of disease models difficult, which have been limited to specific mutations.

Different cell models, including iPSCs and 3D organoids, have been developed to describe the effects of selected LS mutations on early development. These models have proven useful in explaining the physiopathological features of the disease, as well as in drug discovery or as testing platforms for potential metabolic rescue treatments (Ma et al., 2015; Inak et al., 2017; Lorenz et al., 2017). Importantly, compared to 2D differentiation, 3D differentiation provides an improved spatial cellular environment that influences cell fate specification. In this sense, iPSCs have been successfully generated from LS patients harboring different mutations in mitochondrially encoded genes such as ATP synthase membrane subunit 6 (MT-ATP6) (Galera-Monge et al., 2016; Grace et al., 2019), and NADH:ubiquinone oxidoreductase core subunit 3 (MT-ND3) (Hattori et al., 2016), as well as the nuclear-encoded gene surfeit locus protein 1 (SURF1) (Inak et al., 2021). These studies demonstrate bioenergetic defects and disturbances in mitochondrial calcium homeostasis in NPCs carrying mutations associated with LS. Differentiation of LS patient derived iPSCs into neural lineage in both 2D neural cultures and 3D organoids revealed a consistent trend across all LS cerebral organoids, which exhibited impaired growth at various time intervals. Failure to establish proper neuronal wiring and guidance can lead to cognitive and developmental impairments in LS patients. These findings challenge the notion that LS pathology uniquely involves neuronal degeneration and redox imbalance, suggesting instead an impairment of neuronal morphogenesis due to NPC dysfunction.

As described above, during neurogenesis, there is a shift from glycolysis to oxidative mitochondrial metabolism. It is accepted that mitochondrial disorders might arise at the level of NPCs and hinder neuronal morphogenesis. Inak et al. (2021) revealed that SURF1 mutations disrupt metabolic programming in neural progenitor cells (NPCs) preventing the transition from glycolysis to oxidative mitochondrial metabolism crucial for proper neuronal development and morphogenesis. This defect seems specific to neuronal lineage cells, as other cell types carrying the same mutations did not exhibit the same detrimental phenotypes. The authors used differentiated dopaminergic neurons (DN) from patients-iPSC, which are involved in the pathology of LS, and they observed that SURF1 mutant DNs showed a reduced number of neurons, as well as defects in neuronal maturation. SURF1 mutant DNs exhibited reduced sodium and potassium currents compared to CTL DNs, lacked repetitive spiking and postsynaptic currents, and showed an increase in the number non-spiking neurons. Additionally, SURF1 DNs had a reduction of oxygen consumption rate (OCR), maximal respiration, and ATP production rate compared to CTL DNs.

Trying to understand if 3D cerebral organoids can replicate the disruption observed in 2D cultures, Inak et al. (2021) conducted total RNAseq of brain organoids from control and SURF1 mutant groups, identifying gene expression patterns related to morphogens, bioenergetics, proliferation, pluripotency, and neuronal function. SURF1 organoids displayed deregulated expression of morphogens, downregulation of bioenergetic genes (MT-CO3, PDK4, PPARGC1A), and upregulation of glycolytic and proliferative genes. Additionally, genes related to neuronal function were mainly downregulated in SURF1 organoids. Regarding the cytoarchitecture of SURF1 organoids, they show a disorganized neural progenitor pattern and disrupted neuroepithelial layers that confirmed the dysregulation of NPC function and morphogenesis. This dysregulation potentially leads to increased progenitor proliferation and eventual exhaustion of the NPC pool, resulting in impaired neuronal differentiation and reduced organoid size. Overall, authors concluded that due to respiratory defects caused by SURF1 mutations, SURF1 NPCs retained glycolytic and proliferative features, which in turn hampered the establishment of neuronal morphogenesis?

Previous research has also highlighted bioenergetic defects and disturbances in mitochondrial calcium homeostasis in NPCs carrying other mutations associated with LS (Ma et al., 2015; Galera-Monge et al., 2020). Notably, cerebral organoids derived from LS patients with MT-ATP6 mutations exhibited compromised maturation of cortical neurons, resulting in an overall decrease in organoid size, mirroring the reduced size observed in SURF1 organoids (Romero-Morales et al., 2022). It is worth noting that, apart from developmental delays, microcephaly is frequently observed in LS patients (Sofou et al., 2014). Overall, structural alterations found in SURF1 and other LS mutant organoids confirmed the dysregulation of NPCs function and morphogenesis as a relevant mechanism in the pathophysiology of LS.

Romero-Morales et al. (2022) have established LS *in vitro* models by generating and characterizing brain organoids from three commercially available LS fibroblast cell lines and age-matched controls. Characterization of LS iPSCs and 3D differentiation into brain organoids recapitulates the neurological disorders observed in patients, further supporting the utility of brain organoids for research of such mitochondrial diseases. The authors have demonstrated that LS-associated mutations disrupt the mitochondrial network in cerebral organoids, and they suggest that the mitochondrial network in LS lines are more fragmented than in the control, which could be associated with defects in respiratory capacity and developmental disorders. Metabolic analysis of LS organoids revealed lactic acidosis and glycolysis/gluconeogenesis elevated, which would confirm the alterations in mitochondrial reprogramming described during neurogenesis. While the LS organoids showed a reduction in cells positive for upper neural markers, their cellular density remained like controls. Further analysis, using single-cell RNA sequencing or mass cytometry at later maturation stages, may elucidate the effects of LS-associated mutations on cortical cell fate specification. Clinical data from LS patients also highlight marked gliosis as a characteristic finding, potentially resulting from reactive processes secondary to neuronal damage but it could be also a result of an increased propensity of NPCs to differentiate into astrocytes due to LS-causative mutations and mitochondrial dysregulation. It would be important to analyze the gliosis phenotype in more detail and further investigate specific reactivity markers associated with neuroinflammation responses to neuronal damage.

All these models are also helpful to test potential therapeutic strategies for LS, such as gene augmentation therapy or bezafibrate treatment, which promote mitochondrial biogenesis and support neuronal morphogenesis (Lyu et al., 2022). Understanding the metabolic programming of neural progenitors and the role of mitochondrial metabolism in neurogenesis provides insights into the pathology of mitochondrial diseases and offers potential therapeutic avenues for LS, addressing significant unmet medical needs in pediatric patient.

#### 3.1.2 Mitochondrial encephalopathy, lactic acidosis, and stroke-like episodes (MELAS)

Mitochondrial encephalomyopathy, lactic acidosis, and stroke-like episodes (MELAS) is a metabolic disorder typically caused by mtDNA mutations in tRNA genes, predominantly by the A3243G mutation in the *MT-TL1* gene, encoding for tRNA-Leu(UUR) (Yu-Ichi et al., 1990; Nesbitt et al., 2013). The severity of MELAS is influenced by heteroplasmy levels, with symptoms typically manifesting before the age of 20 and more severely in cases of high mutation loads presenting in infancy with developmental delays. While MELAS primarily affects the central nervous system and skeletal muscles due to their dependence on mitochondrial respiration, specific studies on motor neurons or neuromuscular junctions in MELAS have been lacking.

While MELAS is traditionally considered a neurodegenerative disorder, symptoms such as delayed development and growth in early-onset patients or psychiatric conditions in some adult-onset MELAS patients suggest underlying neurodevelopmental deficits. Several groups used iPSCs derived from patients with specific mutations to generate 2D and 3D models. The iPSC-derived neurons with A3243G mutation and various tissues derived from teratomas showed cell-type specific respiratory chain deficiency patterns, with differentiated neurons showing defective mitochondrial complex activity and mitophagy (Hämäläinen et al., 2013). The A3243G mutation was also found to impair human neuronal development and reduce neuronal network activity and synchronicity (Klein Gunnewiek et al., 2020). The MELAS mutation C5541T in the *MT-TW* gene, encoding for TRNA-Trp (UGA/G), also caused cell-type-specific disease phenotypes, with defective neuronal differentiation without apparent impairment of neural progenitors or skeletal muscle cells (Hatakeyama et al., 2015).

While MELAS is traditionally considered a neurodegenerative disorder, symptoms such as delayed development and growth in early-onset patients or psychiatric conditions in some adult-onset MELAS patients suggest underlying neurodevelopmental deficits. Several groups used iPSCs derived from patients with specific mutations to generate 2D and 3D models. The iPSC-derived neurons with A3243G mutation and various tissues derived from teratomas showed cell-type specific respiratory chain deficiency patterns, with differentiated neurons showing defective mitochondrial complex activity and mitophagy (Hämäläinen et al., 2013). The A3243G mutation was also found to impair human neuronal development and reduce neuronal network activity and synchronicity (Klein Gunnewiek et al., 2020). The MELAS mutation C5541T in the *MT-TW* gene, encoding for TRNA-Trp (UGA/G), also caused cell-type-specific disease phenotypes, with defective neuronal differentiation without apparent impairment of neural progenitors or skeletal muscle cells (Hatakeyama et al., 2015).

Winanto et al. (2020) derived iPSCs from a patient with severe MELAS and an isogenically corrected control to investigate motor neuron deficits. The MELAS iPSCs were able to differentiate into NPCs, but the generation of motor neurons was severely compromised. While motor neurons were not viable in conventional 2D protocols, they could be reproducibly generated in the organoid model, highlighting the utility of neural organoids. They demonstrated that MELAS organoids fail to properly differentiate into motor neurons, as indicated by the high expression of key markers for motor CNPs, such as OLIG2 and SOX1, which persist even on day 42 of differentiation. This work does not provide details about the lack viability of motor neurons in 2D-cultures; however, these authors conclude that deregulated Notch signaling could explain the neurogenesis defects in MELAS. Is it possible that these pathophysiological mechanisms are more prominent in 2D-cultures due to the lack of cell-cell communication. The study found defects in mitochondrial respiration, either through the mtDNA A3243G mutation, or through inhibition of the mitochondrial Complex I, leading to elevated Notch signaling and demonstrated that inhibition of Notch pathway corrected neurogenesis defects in MELAS spinal organoids. This hyperactive Notch signaling, previously identified as a cause of delayed neurogenesis (Berezovska et al., 1999; Levy et al., 2002), has been demonstrated to be a mechanism implicated in the disease and suggests a novel mitochondrial-Notch crosstalk previously unrecognized in MELAS.

Thus, organoids have provided insights into the complex pathophysiology of MELAS, indicating that neurodevelopmental defects are intrinsic to MELAS. This suggests that motor deficits in MELAS patients result from a combination of muscular and motor neuron pathologies. Similarly, neurodevelopmental defects in cortical neurons could explain developmental delays and psychiatric manifestations observed in some MELAS patients.

In addition, spinal organoids facilitated the investigation of potential therapeutic targets for further exploration (Winanto et al., 2020). In this context, treatment with the gamma secretase inhibitor DAPT in MELAS organoids demonstrated the ability to reverse neurodevelopmental and neurite outgrowth defects, highlighting the potential therapeutic target of Notch signaling modulation to ameliorate neurodevelopmental deficits associated with the disease.

#### 3.1.3 Myoclonic epilepsy and ragged-red fibers (MERRF)

Myoclonus Epilepsy with Ragged-Red Fibers or MERRF syndrome is characterized by symptoms such as myoclonus epilepsy, muscle weakness, atrophy, cerebellar ataxia, hearing impairment, and cognitive decline (Shoffner and Wallace, 1992). The clinical presentation of MERRF can exhibit significant variation, even among individuals within the same family. Similar to MELAS, MERRF is caused by mutations in transfer RNA (tRNA) genes, being one of the most common the A8344G mutation in the *MT-TK* gene, coding for tRNA-Lys.

The mechanism by which this tRNA(Lys) mutation causes mitochondrial dysfunction in cardiomyocytes or neurons remains unclear. To date pathophysiological alterations have been investigated by developing iPSCs from MERRF patients and isogenic controls and subsequent differentiation into cardiomyocytes and neural progenitors. These models have shown that cells with A8344G mutation exhibit reduced oxygen consumption, increased ROS production, altered growth, and fragmented mitochondria indicating mitochondrial dysfunction in MERRF syndrome (Chou et al., 2016). In the future, the development of cerebral and other types of 3D organoids will likely provide new opportunities for *in vitro* modeling of this disease. In this regard, 3D organoids will offer a more accurate representation of the human brain compared to traditional 2D cell cultures. By analyzing MERRF patient-derived organoids, it will be possible to investigate how mitochondrial dysfunction affects neuronal maturation induced by A8344G mutation or others. This will allow for a more faithful modeling of this disease and gain insights into the underlying molecular mechanisms caused by mitochondrial dysfunction and gain insights into underlying molecular mechanisms produced by mitochondrial dysfunction and altered redox state with reactive oxygen species (ROS) overproduction found in 2D models (Villanueva-Paz et al., 2019), and how these alterations might affect cell fate and interactions between neuronal populations. Further, organoids allow screening potential drug candidates in a more physiologically relevant environment, accelerating the drug development process.

#### 3.1.4 Leber’s hereditary optic neuropathy (LHON)

Leber’s Hereditary Optic Neuropathy, LHON is a rare genetic disorder that primarily affects the eyes, specifically the optic nerve, responsible for transmitting visual information from the eye to the brain. LHON is caused by mutations in the mitochondrial DNA, particularly in genes encoding the subunits of complex I of the mitochondrial electron transport chain such as *MT-ND1*, *MT-ND4*, and *MT-ND6*. These mutations lead to mitochondrial dysfunction and impaired energy production, resulting in the death of retinal ganglion cells and optic nerve damage. The condition results in severe visual impairment or blindness, usually the only clinical manifestation of the disease.

To produce models to investigate LHON, researchers have generated iPSCs with mutations in the *MT-ND4* gene (G11778A) to differentiate neurons and retinal ganglion cells (Yang et al., 2020; Danese et al., 2022). The retinal ganglion cells displayed elevated oxidative stress and apoptosis linked to abnormal mitochondrial transport (Yang et al., 2019), disturbed action potentials (Yang et al., 2019), as well as impaired neurite extension and increased mitochondrial biogenesis as a compensatory strategy to counteract the defective phenotype induced by LHON mutations (Wu et al., 2018).

#### 3.1.5 Mitochondrial neurogastrointestinal encephalomyopathy (MNGIE)

Mitochondrial neurogastrointestinal encephalomyopathy (MNGIE) is a rare autosomal recessive disease caused by pathogenic variants in the nuclear *TYMP* gene encoding the thymidine phosphorylase enzyme (Nishino et al., 2000). Thymidine phosphorylase has a pivotal role in the nucleoside salvage metabolic pathway that is critical for the maintenance of the mitochondrial genome. Consequently, loss of thymidine phosphorylase activity results in the accumulation of mutations in the mtDNA, which ultimately leads to the failure of mitochondria to perform oxidative phosphorylation. The disease is characterized by severe gastrointestinal dysmotility, sensorimotor peripheral neuropathy, severe muscle weakness, and progressive leukoencephalopathy, with patients usually dying from a combination of nutritional failure and muscular disability.

Murine models based on Tymp^–/–^/Upp^–/–^ genes double knock-out have been developed to study MNGIE but displayed inconsistencies with the clinic likely due to differences in the deoxyribonucleoside metabolism between humans and mice (Haraguchi et al., 2002). With the hope of overcoming this limitation, iPSCs have been recently derived from a patient with MNGIE and differentiated into cerebral organoids, characterized by the presence of diverse cell types including glial and neuronal cells (Pacitti and Bax, 2018). Further characterization will be needed to evaluate the utility of the model for studying MNGIE.

### 3.2 Syndromes caused by expansion of DNA

#### 3.2.1 Friedreich’s ataxia

Friedreich ataxia (FRDA) is a rare autosomal-recessive neurodegenerative and cardiac disorder characterized by progressive limb and gait ataxia, dysarthria, dysphagia, loss of deep tendon reflexes, oculomotor dysfunction, and cardiomyopathy, with additional symptoms like diabetes mellitus and defective hearing in some cases. It occurs due to silencing or severe reduction of transcription of the *FXN* gene, encoding a mitochondrial protein which belongs to the FRATAXIN family, caused by the expansion of GAA trinucleotide repeats within its first intron. This expansion leads to repressive histone modifications around the repeat tract, contributing to gene silencing. Frataxin plays a crucial role in iron metabolism and respiration, with insufficient levels resulting in mitochondrial dysfunctions affecting cellular integrity (Pastore and Puccio, 2013).

FRDA iPSC-derived neurons have unaltered ability to differentiate into neuronal cells although showing some disease hallmarks, including delayed maturation and mitochondrial dysfunction (Hick et al., 2013; Maheshwari et al., 2023). Recently, a more precise 3D model involving iPSC-derived sensory neurons within the dorsal root ganglia (DRG) structure has been developed to better understand FRDA pathology. Mazzara et al. (2020) investigate FRDA by differentiating iPSCs into organoids that closely resemble the geometry of somatic dorsal root ganglia. This study establishes a patterned iPSC-derived sensory neuronal circuitry between DRG organoid (DRGO) sensory neurons and muscle intrafusal fibers, offering an improved *in vitro* model for studying FRDA pathology and investigating potential therapeutic interventions, such as CRISPR/Cas9-mediated removal of trinucleotide expansions in the *FXN* gene intron. These 3D organoids contain principal sensory neural subtypes and glial cells found in somatic DRGs. Single-cell RNA sequencing analysis revealed the assembly of sensory neurons and their precursors at various differentiation stages within the DRG organoids, with nociceptors being the least represented subtype. Despite the prolonged *in vitro* maturation process, mature sensory neurons within the DRG organoids exhibited extensive functional features, including excitability, synaptogenic competence, and the ability to form complex structures like muscle spindles. FRDA patient-derived DRG organoids exhibited impaired survival rates of sensory neurons and various morphological and molecular defects in both cytosolic and mitochondrial compartments due to FXN silencing. The DRG organoids are a robust human *in vitro* tissue model demonstrating stability, reproducibility, and capability to recapitulate pathological phenotypes associated with FRDA.

Current therapeutic approaches for FRDA, including HDAC inhibitors and gene replacement therapy, are under development, with promising results reported in preclinical models, but their efficacy in rescuing FRDA phenotypes remains to be fully determined. Moreover, targeted *FXN* intron 1 genomic deletions in patient iPSCs effectively ameliorated the molecular and cellular disease phenotypes in FRDA DRG organoids. The complete removal of intronic regions flanking the GAA repeats showed the most significant restoration of FXN expression, compared to elimination of only the GAA tract, offering a potential therapeutic strategy for FRDA. Overall, the developed DRG organoid system represents a valuable platform for modeling and testing therapeutic interventions for FRDA and other pathologies affecting the peripheral nervous system.

### 3.3 Syndromes caused by mtDNA large-scale deletions

#### 3.3.1 Mitochondrial DNA depletion syndrome (MDS)

Mitochondrial DNA depletion syndrome (MDS) is a group of severe, phenotypically heterogeneous, recessively inherited disorders characterized by marked reduction of the mtDNA content leading to impaired energy production in the affected organs. MDS results in affected muscle, liver, or/and brain, depending on the mutated gene. Most common form of MDS is associated with mutations in the *DGUOK* gene, encoding the mitochondrial deoxyguanosine kinase, that primarily affect the brain and the liver (Mandel et al., 2001). DGUOK mediates the phosphorylation of deoxyguanosine and deoxyadenosinepurine into the corresponding nucleotides in mitochondria and its deficiency creates an imbalance in the mitochondrial dNTPs pool that affects mtDNA synthesis. There is no current therapy for this disease and patients commonly die from liver failure around 2 years of age, after developing iron overload that leads to ferroptosis in this organ.

Guo et al. (2021) established an *in vitro* liver disease model of liver organoids and hepatocytes derived from iPSCs of *DGUOK* mutant patients to unlock the molecular cause of iron overload. Using this model, authors show that mtDNA depletion leads to mitochondrial dysfunction, reduced ATP production and ROS enhancement, which causes glutathione exhaustion, a known cause of ferroptosis. Importantly, the effects could be reduced by supplying the glutathione precursor, N-acetylcysteine (NAC) offering a promising therapeutic strategy for these patients.

#### 3.3.2 Chronic progressive external ophthalmoplegia (CPEO)

*POLG1* is a nuclear gene that encodes mitochondrial DNA polymerase gamma, responsible for the synthesis of mtDNA. Pathogenic variants in *POLG1* cause progressive external ophthalmoplegia (PEO) characterized by weakness of the eye muscles accompanied by other more variable symptoms. These variants have also been associated with the development of symptoms of Parkinson’s Disease in PEO patients (Reeve et al., 2013).

In an effort to understand how the loss of *POLG1* function causes Parkinsonism, Chumarina et al. (2019) generated a model of midbrain dopaminergic neuron-containing spheroids from a patient with PEO and Parkinsonism. This model allowed them to identify altered inflammatory signaling and increased glycolysis in the patient derived spheroids.

#### 3.3.3 Kearns-Sayre Syndrome (KSS)

Kearns-Sayre Syndrome (KSS) is a rare neuromuscular disorder with onset usually before the age of 20. KSS is a severe syndromic variant of CPEO that progresses to multisystemic effects due to large deletions in the mtDNA of unknown cause. Intriguingly, the mtDNA deletions in patients with KSS are largely found in muscle tissue but are predominantly absent in blood cells (Holt et al., 1988). Taking advantage of this fact, Sequiera et al. (2021) derived iPSCs from blood mononuclear cells of two KSS patients and found that cells could be differentiated into neural progenitors, fibroblast-like cells, and cardiomyocytes without signs of mitochondrial defects. This strategy might provide a source of healthy cells for personalized autologous cell replacement therapies in these patients.

#### 3.3.4 Pearson Marrow-Pancreas Syndrome (PMPS)

Pearson Marrow-Pancreas Syndrome (PMPS) is a congenital life-threatening bone marrow failure disorder caused by large deletions in mtDNA of unknown cause. Deleted mtDNA in patients’ cells exists in varying proportions relative to normal mtDNA, a mixture termed heteroplasmy.

Cherry et al. (2013) assessed the heteroplasmy issue by generating several iPSCs clonal lines from a PMPS patient. Heteroplasmy varied among iPSCs clonal cell lines and throughout culture time, which allowed isolating pluripotent cells with different percentages of mutant mtDNA. Differentiation of iPSCs cell lines with the highest mutant mtDNA burden yielded erythroid cells with inappropriate iron deposits that are characteristic of PMPS. Additionally, similar to Sequiera et al. (2021) they were able to generate iPSCs from KSS patients’ skin-derived fibroblasts without mtDNA alterations. Therefore, both papers demonstrated that it is possible to derive disease-free patient-specific iPSCs lines from patients with a low burden of a mosaic genetic abnormality.

Research on drug discovery and therapeutic development in brain organoids modeling mitochondrial-based neurodevelopmental disorders is currently limited. Most progress has been made using 2D models, as previously mentioned. Reproducing complex 3-D structures derived from patients in the laboratory and overcoming challenges associated with their generation, manipulation, and analysis would enable us to understand the effect of therapeutic compounds on different types of neurons and cellular populations of the nervous system and it would also allow us to investigate the role of the inflammatory/immune system in a more disease-specific and patient-transferable manner. Establishing an adequate vascularization system and blood-brain barrier is crucial for understanding the mechanisms of these systems on the delivery of therapeutic compounds in the CNS. All these advances could also lead to the development of specific and more efficient nanoparticles and gene therapies for the central nervous system.

## 4 Brain organoids: screening or drug repurposing in models of mitochondrial diseases

One of the more relevant questions for the researchers is whether organoids might replace or reduce the use of animal models in drug screening and cancer immortalized cell lines in high-throughput screening (HTS) (Entzeroth et al., 2009). The generation of organoids to model diseases and their use in screening drugs or cell therapy represent a great expectation for researchers and patients (Costamagna et al., 2021; Lampart et al., 2023). However, although in the last 10 years many new drugs have been developed to treat different human diseases, in the field of mitochondrial pathologies with neurodevelopmental disorders, the discovery of new compounds or treatments has not been very effective. Despite efforts made by the scientific community, it is estimated that 95% of drugs fail clinical trials (Wong et al., 2019). Disease models that recapitulate pathophysiological mechanisms and phenotypes of mitochondrial and neurodevelopmental disorders remain a fundamental bottleneck in order to identify new compounds and successfully translate them to clinical practice.

In this section, we review the main limitations and advantages of using brain organoids in high-throughput screening (HTS) or drug repurposing (DR) to discover new treatments for mitochondrial diseases that cause neurodevelopmental disorders.

Studies about HTS or DR in brain organoid models for neurodevelopmental diseases and mitochondrial disorders have not yet been reported, which to some extent reflects the enormous difficulty and limitations of brain organoids in modeling these groups of diseases and their applications in drug discovery.

**FIGURE 4:**
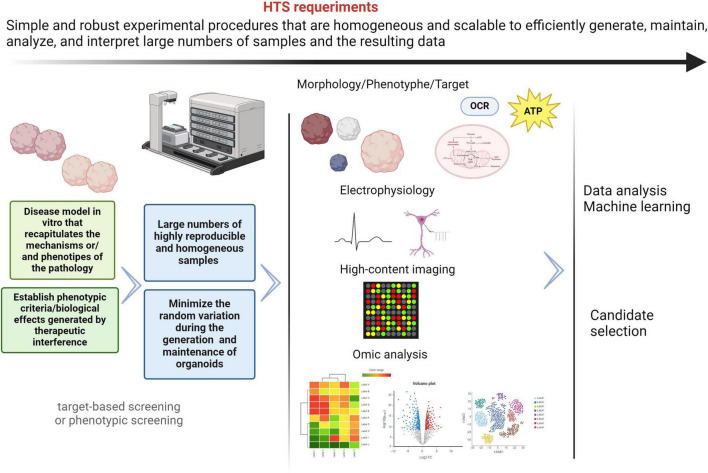
General HTS requirements. The different processes included in HTS must be simple, scalable and robust, in order to obtain reliable and homogenous results. The use of brain organoids derived from patients with mitochondrial diseases and neurological manifestations represents an important challenge to drug discovery in HTS platforms. Despite the current limitations, innovations in protocols, instrumentalization/automation, and omics analysis techniques are enabling exciting advances in the field of drug discovery. Nevertheless, these approaches have not been sufficiently explored and neurodevelopmental diseases model with mitochondrial involvement. Created with BioRender.com.

**High-throughput screening** (**HTS**) is an automated/robotic system to test up to hundreds of thousands or even more compounds *in vitro*, to identify new potential drugs (Cota-Coronado et al., 2020; Durens et al., 2020). This system is one of the most efficient and quick processes and provides starting points for drug design. In general terms, the main requirements for HTS assays are summarized in Figure 4.

Traditionally, drug discovery and HTS assays rely on cancer immortalized cell lines, whose energetic requirements are less dependent on oxidative mitochondrial phosphorylation (OXPHOS) (Horvath et al., 2016). Furthermore, this cellular model lacks functional and phenotypical properties of neuron cells and the brain. Genetic and epigenetic abnormalities resulting from long-term *in vitro* culture in such an unphysiological environment also contribute to substantial changes in the physiological characteristics of immortalized lines. This has an impact on the heterogeneity of cultures and results between passages and laboratories, in the same cell line. Therefore, they are not an effective model for drug discovery model for mitochondrial pathologies. On this basis and considering that brain organoids are 3D cytoarchitectures resembling the embryonic human brain, many researchers and companies are developing protocols and pipelines for the generation, maintenance and analysis of brain organoids to model neurodevelopmental diseases (Lee et al., 2017; Yang et al., 2023; Zhou et al., 2023). However, it is necessary to address new technical approaches that allow us to evaluate the mitochondrial function and structure in these 3D models.

There is evidence that the response to therapy in patients correlates with effects in corresponding patient-derived organoids, such as pancreatic, ovarian, uterine, colon, gastrointestinal, brain, and cancer (Vlachogiannis et al., 2018). In addition, the beneficial effects of drugs, such as PTC-124 and Ataluren, in animal models of cystic fibrosis were not demonstrated in human intestinal organoids, which is consistent with the results in two-phase clinical studies. Although there are still challenges to overcome, patient-derived organoids represent a potential preclinical model (Zainal Abidin et al., 2017). The power of HTS in organoid-based drug discovery have been highlighted in cancer and to lesser extent in neurodegenerative diseases, such Alzheimer’s and Parkinson’s (Krall et al., 2020; Park et al., 2021; Renner et al., 2021; Jeong et al., 2023). Nevertheless, based on reports, neurodevelopmental disorders with mitochondrial involvement are not being covered sufficiently.

HTS assays can be target- or phenotypic-based screening. For most rare diseases, pathological mechanisms and molecular targets are unknown, so target-based screening may be impossible. Phenotypic screening results are more attractive for this kind of disease, since it only requires the monitoring of some specific biological functions after compounds administration (Khurana et al., 2015). In an organoid-based HTS assay, it is crucial to establish the phenotypic criteria to classify the control and pathological conditions. These phenotypic features must be measurable, easily detected, in addition to matching the clinical symptoms. Ultimately, viability and metabolic activity are two of the most important measurable parameters in HTS assays in different organoids, but the information obtained from such measurements is very limited. Among the parameters or readouts to establish the phenotypic deficits for mitochondrial and neurological disorders are organoid size, cell migration, gene expression, cortical lamination, neurogenesis, axonal growth, electrophysiological parameters, oxidative stress, oxygen consumption or ATP production. Some authors have shown that parameters of mitochondrial functions used in 2D cultures, specifically ATP production, can be measured in a 96-well plate in neuronal organoids. Oxidative stress and mitochondrial potential measurements have also been assayed in 96-well plates, in this case in retinal organoids (Nzou et al., 2018; Le et al., 2021; Ludikhuize et al., 2021).

Currently, protocols for the generation of 3D models have advanced remarkably; however, their use in high-throughput drug screening is still limited, especially for neurodevelopmental and mitochondrial diseases, as mentioned above. Complexity of mitochondrial diseases causing neurodevelopmental disorders, lack of profitability of industry derived from the low frequency of these diseases and the difficulty that still exists in developing brain organoids reproducible, reliable and scalable are some of the limitations. Although innovations such as vascularization, genome editing, omics-techniques, high-content imaging (HCI), machine-learning-based and automated systems are already a reality in organoids, protocols need to continue evolving to improve tissue communication, cell patterning signals, body axis simulation and spatial-temporal control of differentiation (Lokai et al., 2023). Engineering and instrumentalization advances to reconstitute brain-mimetic microenvironments will improve the development of efficient and reliable platforms to model and drug discovery of human brain diseases (Patel et al., 2024). Organoid on-chips are microfluidic chamber devices that provide a periodic flow and mimic the cerebrospinal and interstitial spaces. Brain organoids can be inserted into these chips, facilitating the oxygen supply and nutrient/waste exchange by controlling the environmental conditions of brain organoids (Cho et al., 2021; Zhou et al., 2021; Saorin et al., 2023). Microfluidic chips lead to a significant reduction of cell death and consequently an improvement of maturity, although their use is not enough extended.

Aspects such as production efficiency and organoid homogeneity, but also culturing conditions and handling are important challenges in drug screening. Optimization and standardization of reliable protocols are crucial to obtain meaningful results. Renner et al. (2020) have design an automated HTS workflow in 96-well formats (“automated midbrain organoids,” AMOs) to generate and analyze human midbrain organoids. Automation of all processes, from generation, maintenance, immunostaining, tissue clearing and HC imaging, allows scaling and homogenization of brain organoids, as well as drug testing (Renner et al., 2020). High-throughput screening (HTS) assays require the generation, maintenance, analysis, and interpretation of a large number of samples. Consequently, the analysis of data and results is complex and time-consuming. Fortunately, the progression of artificial intelligence (AI) tools already allows us to process and classify large datasets, as well as correlate results obtained even from different areas (Renner et al., 2021; Metzger et al., 2022).

**Drug repurposing or drug repositioning** is defined as the discovery of new applications of approved or investigational compounds. This process saves time, and it is more cost-effective than the development of new orphan treatments, representing a significant advantage for rare diseases and patients (Shah et al., 2021; Kakoti et al., 2022). However, in many areas of research, repurposed drugs may not be effective in clinical trials or may not maintain the benefit-injury equilibrium (Fetro and Scherman, 2020). Drug repurposing has been used for years, but new approaches have been developed to make them more systematic and efficient. As we mentioned before, drug screening in organoid models has been developed on a larger scale for diseases such as cancer, and other common pathologies (Mao et al., 2023; Mertens et al., 2023). Recently, Spelier et al. (2023) have developed a drug repurposing assay, in a 384-well screening format, using cystic fibrosis patient-derived intestinal organoid.

Another aspect to consider is the legal and regulatory requirements, which should be carefully analyzed before any study of drug repurposing. Computational drug repurposing, because of the progression in HTC technologies, and omics-based data related to disease indications, are considered as cutting-edge techniques for drug discovery (Emon et al., 2017; Paranjpe et al., 2019; Hendrickx et al., 2020). We wanted to focus on the use of these methodologies for drug discovery in rare diseases and particularly neurological diseases with mitochondrial alterations (Hodos et al., 2016). While these disciplines continue to advance, specific experimental and economic barriers arising from these complex diseases need to be overcome.

## 5 Advantages and limitations of brain organoids for drug screening of mitochondrial diseases with neurodevelopmental manifestations

### 5.1 Advantages

• Restore the lack of physiological neural models:
  ○ Recapitulate human neural networks, functions and cytoarchitecture of specific regions of the brain.
  ○ Recapitulate the first stages of fetal brain development. Most mitochondrial dysfunctions affect the early stages of brain embryonic development or perinatal stages.
• Closer to patient’s physiology and genetic background.
  ○ Recapitulate neural phenotypes of diseases.
  ○ Recapitulate mitochondrial phenotypes of diseases.
• Valid for drug screening and personalized medicine.
• More efficient preclinical model. Results are easier translated to the patients.
• Less ethical concern than animal or mammalian models.
• Consensus of the scientific community.

### 5.2 Limitations

#### 5.2.1 Limitations due to the model

• The human brain dramatically outperforms current state-of-the art of brain organoids models. It contains 86 billion interconnected neurons capable of storing and processing large amounts of information in an extremely efficient manner.
• Sample heterogeneity and lack of reproducibility intra- and inter-laboratory.
  ○ Inherent variability within biological samples:
• Environmental and genetic factors.
• Variable ability of different iPSC lines to generate relevant architectures and cell types.
• Most organoid protocols rely on self-organization, rather than a regulated spatial arrangement.
• Low standardization of protocols.
  ○ A multitude of reagents and different protocols across experiments were used to generate patient-iPSC and organoids.
  ○ Use of different extracellular matrix-like structures. Physiologically, microglia and vascular endothelial cells contribute to ECM formation and maintenance. However, this type of cell may lack current neural organoids. Examples of ECM-like structures are:
• MATRIGEL, which is the most widely used, but its composition is not fully characterized.
• ECM derived from fetal porcine brains combined with silk scaffolding (Sood et al., 2019).
• Polylactic-co-glycolic acid (PLGA) molecules (Bishop et al., 2017; Knight et al., 2018).
• Differences in the degree of maturity.
  ○ Lack of spontaneous vascularization, which has two main effects: hypoxia and limited nutrient diffusion. These factors affect survival and degree of maturity that brain organoids can achieve and are a limiting factor, especially in modeling adult-onset diseases. Nevertheless, in mitochondrial dysfunctions that affect the early stages of brain embryonic development or perinatal stages, maturity may not become a barrier. Currently, different groups are developing methods to overcome these issues based on the incorporation of vascular endothelial growth factor (VEGF) and WNT7b or co-culture methods with mesodermal progenitors generating a vascular networks (Shi et al., 2020; Strobel et al., 2022; Ye, 2023). These protocols have shown a cross-talk between neurons and endothelial cells, as well as control the diffusion of oxygen and nutrients. Blood-brain barrier (BBB) is responsible for filtering the blood of toxic compounds to the brain and some drugs. BBB represents a challenge for drug delivery or neurotoxicity evaluation into the brain organoids and it must be taken into account in drug discovery workflows (Sokolova et al., 2020; Kofman et al., 2022).
• Limited scalability.
  ○ HTS requires automated and robotic systems, which are often not available for most research due to their complexity and expense. Drug screenings are generally performed in 384 or 1536-well plates, which makes it difficult to grow and maintain brain organoids due to their size and cell number.

The limitations described above can result in variable or low penetration of small molecules or other compounds. This can lead to poor or weak drug effects and readouts in different cell types or layers into the organoid.

#### 5.2.2 Limitations due to the disease

• The pathophysiology of mutations or mechanisms underlying the neuronal pathology of mitochondrial diseases is still unknown in most patients, which makes it difficult to interpret the mitochondrial or neural readout in pharmacological screenings.
• Rare diseases. Low frequency of mutations. Reduced number of patients with the same mutations, limiting the statistical results.
• Complex and multisystemic clinical manifestations.

## 6 Final conclusions

Advancements in the generation of brain organoids represent a revolution in the research of mitochondrial diseases affecting neurodevelopment. However, despite the increasing number of brain organoids for modeling rare diseases, the techniques for metabolic and structural analysis of mitochondria have not yet been sufficiently developed. Indeed, most readouts in these 3D models are based on structural characteristics.

There is a promising journey ahead. Brain organoids fill a significant gap in research of human neurological disorders. Nevertheless, there are several theoretical and practical limitations to understanding the role of mitochondria in the development of the neurological system and the progression of diseases. Currently, there is a lack of precise information about patterns of neural maturation in humans, and the pathophysiological mechanism underlying most mitochondrial diseases affecting the nervous system remains unknown. Regarding practical obstacles, heterogeneity of size and morphology, survival, handling and scalability stand out. Some of these limitations may be overcome by the unification of protocols and the adoption of engineering solutions, automated liquid handling and high content analysis. These factors are essential for drug screening. Current studies have focused on structural readouts, such as survival and neural differentiation, gene expression, size, cortical lamination, etc. However, aspects such as communication between different neural cell types and vascularization are being addressed from various scientific and technological fields.

The cooperation between private companies and researchers of different disciplines, as well as discussions on these approaches, are essential for the use of brain organoids as a proper model of mitochondrial diseases and neurodevelopmental disorders.

## Author contributions

RC: Writing – original draft, Writing – review and editing. EG-M: Writing – review and editing. ES: Writing – review and editing. MB: Writing – original draft, Writing – review and editing. BM-D: Writing – original draft, Writing – review and editing. CS-O: Writing – review and editing, Funding acquisition, Supervision. IL: Writing – original draft, Writing – review and editing. MC-A: Writing – original draft, Writing – review and editing.
